# Full Mouth Reconstruction of a Skeletal Class II Division 1 Patient with Adenoid Cystic Carcinoma Using an Interim Immediate Obturator and a Definitive Obturator

**DOI:** 10.1155/2017/5458617

**Published:** 2017-04-04

**Authors:** Mehran Bahrami, Seyed Mehran Falahchai

**Affiliations:** Dental Research Center, Department of Prosthodontics, Faculty of Dentistry, Tehran University of Medical Sciences, Tehran, Iran

## Abstract

A 61-year-old female patient with adenoid cystic carcinoma (ACC) of the right maxilla and Angle class II division 1 malocclusion had received a subtotal maxillectomy in right side and used a conventional clasp-retained obturator. After implants placement, a maxillary interim immediate obturator (IIO) and then a definitive obturator using six endosseous implants were fabricated. During one-year follow-up, the patient was completely satisfied. Ideally, after implants placement in edentulous patients suffering from hemimaxillectomy, an implant-supported obturator (ISO) is designed in order to prevent nasal reflux and to improve speech and swallowing. However, in the following case, because of skeletal class II division 1 malocclusion and implants insertion in the premaxilla, using an ISO was impossible because it would cause excessive upper lip protrusion and lack of anterior teeth contact. Therefore, a five-unit implant-supported fixed partial denture (FPD) was fabricated in the maxillary anterior segment so that anterior teeth contacts were possible and the patient's normal lip support was achieved. A bar and three ball attachments were used in the maxillary posterior segment. A closed-hollow-bulb ISO was preferred. Conventional ISO in these patients results in several problems. Using a maxillary anterior FPD along with ISO caused satisfactory results in the current patient.

## 1. Introduction

Adenoid cystic carcinoma (ACC) is considered as an uncommon tumor. It includes about 1% of head and neck malignancies and about 10% of all salivary glands tumors. ACC is the most common minor salivary glands tumor. Radical surgical resection and postoperative radiotherapy are the gold standard treatment of ACC [1].

Maxillary defects diminish patient's quality of life significantly [2]. In these cases, obturator prosthesis is often the chosen treatment option because of difficulty in surgical reconstruction. Maxillofacial prosthesis is still indicated in most maxillectomy patients, after surgical reconstruction [3]. The most important aim of prosthetic rehabilitation is the preservation of residual teeth and tissues and separation of nasal and oral cavities [4].

Conventional obturators have more movements in edentulous patients. Retention, support, and stability of maxillary obturator will be improved by using osseointegrated implants in residual maxilla [2]. The most suitable place for insertion of implants is the anterior segment of maxilla and tuberosity [5].

Roumanas et al. [6] found that survival rate of implant is 63.6% in irradiated patients who were subjected to average dose of 50 Gy and is 82.6% in nonirradiated patients. Anterior implants have 2.7 times more exposed threads than posterior implants.

The aim of the current case study was to fabricate a maxillary interim immediate-obturator (IIO) and a definitive obturator (DO) using endosseous implants and a five-unit anterior fixed partial denture (FPD) in a patient with ACC and Angle class II division 1 malocclusion. In order to achieve anterior teeth contact and the normal support of the lip, a five-unit implant-supported fixed partial denture (FPD) was fabricated in the maxillary anterior segment.

## 2. Case Presentation

**Figure 1:**
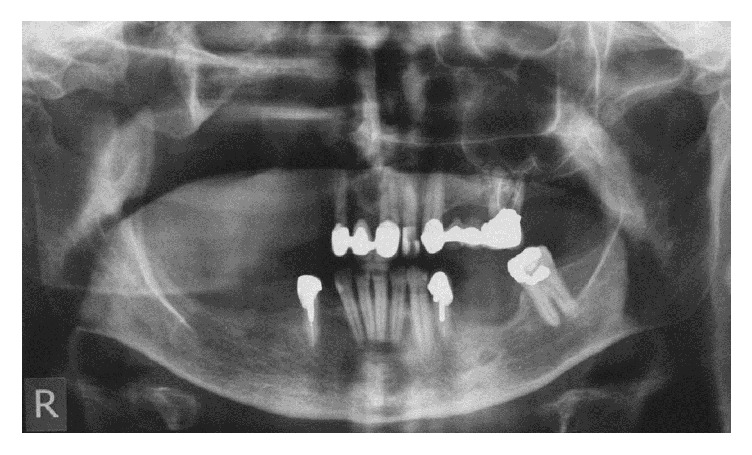
Patient's preoperative panoramic view.

**Figure 2:**
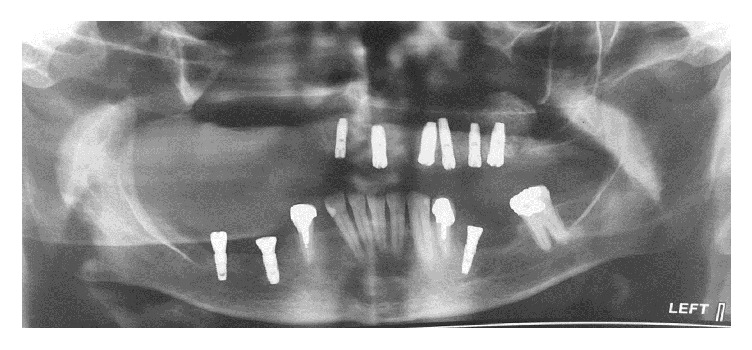
Panoramic view after implant insertion.

**Figure 3:**
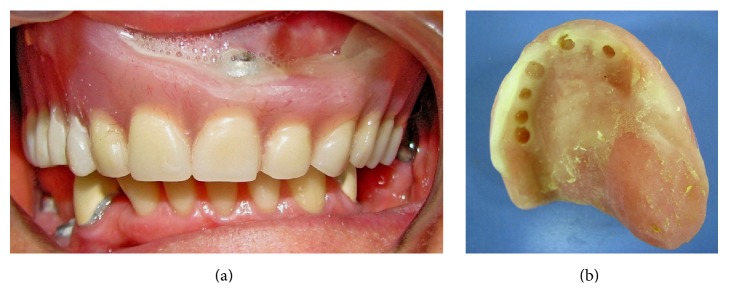
(a): Intraoral view of the interim immediate obturator (IIO) with tissue conditioner (TC) applied. (b): intaglio surface of the IIO.

**Figure 4:**
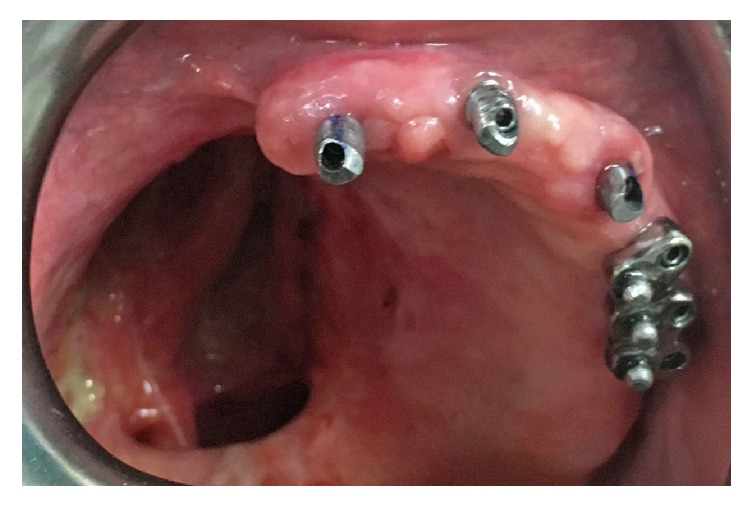
15° angled abutments and bar and balls were placed and torqued.

**Figure 5:**
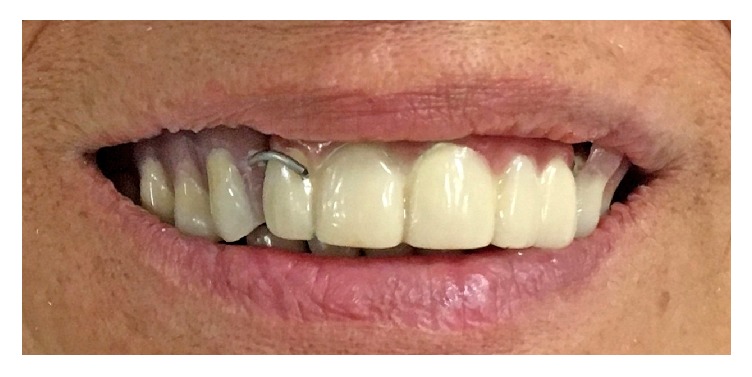
Delivery of the maxillary definitive obturator (DO) and 5-unit FPD.

**Figure 6:**
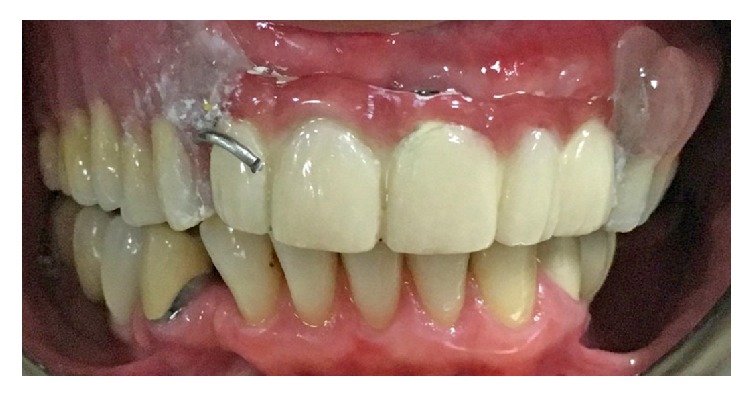
TC was applied to the tissue surface of the DO in order to obtain the optimal peripheral seal.

**Figure 7:**
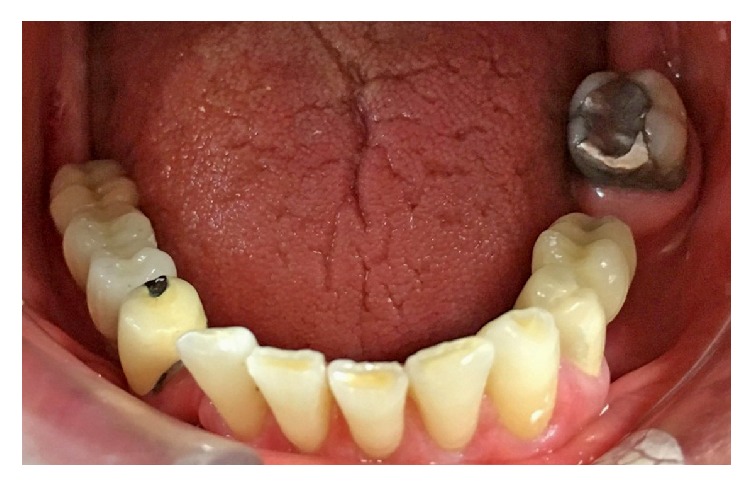
Delivery of mandibular-implant-supported restorations.

**Figure 8:**
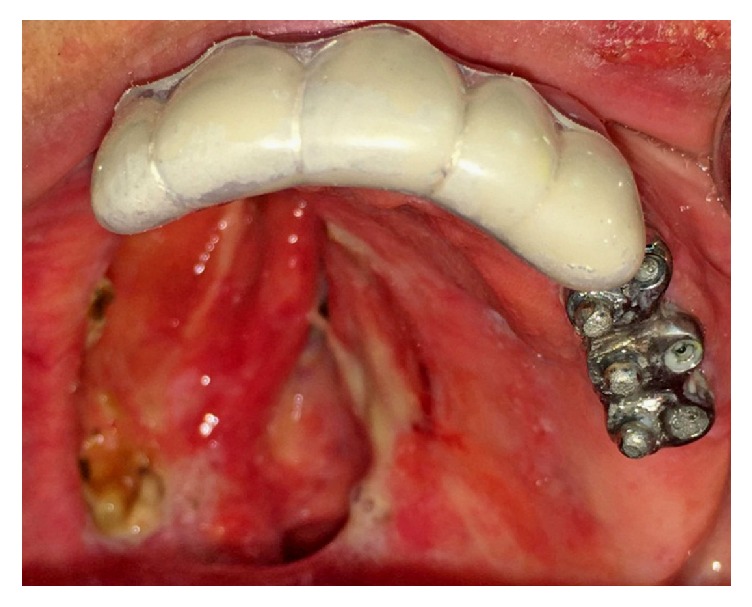
Delivery of the soft guard to protect the maxillary 5-unit FPD.

**Figure 9:**
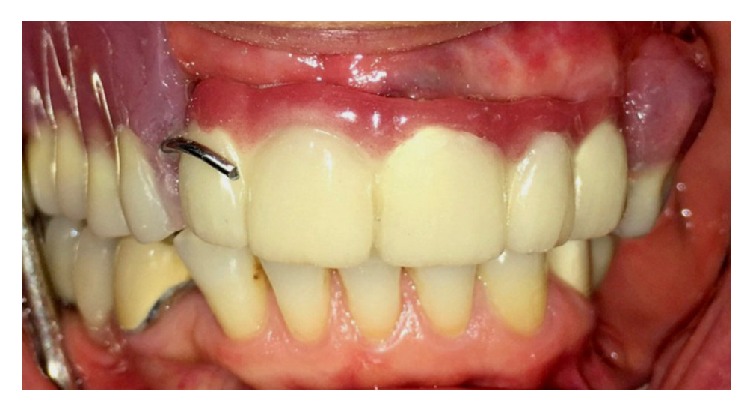
DO with TC was processed again and delivered.

A 61-year-old female patient with ACC of the right maxilla was referred to the Implant Department in Tehran University of Medical Sciences. The patient had received a subtotal maxillectomy in right side and used a conventional clasp-retained obturator. There was no need for radiotherapy according to the surgery team. The remaining teeth in maxilla included #7, #9, #10, #11, and #15. Clinical and radiographic examination of maxilla revealed a three-unit FPD with right lateral incisor and left central incisor retainers, a four-unit FPD with left canine and left second molar retainers, two post and core crowns on first premolars, and an extensive amalgam restoration on left second molar. All maxillary teeth had unfavorable C/R ratio and were hopeless according to severe caries and severe periodontitis (Figure 1). So the clinical decision for the maxilla was extraction of all remaining teeth and insertion of six implants (Figure 2). In order to fabricate a maxillary IIO, a pick-up impression was taken with the existing obturator with irreversible hydrocolloid (Kerr, USA) prior to implant surgery. The teeth were removed from the cast (cast surgery) and acrylic denture teeth (Myerson, Trinidad and Tobago) were replaced. Teeth arrangement was approved by patient on the articulator. IIO was processed to use immediately after teeth extraction. In implant surgery appointment, open sinus lift was performed and six fixtures were inserted in maxilla using two-stage surgery in the place of #7 (Dentium/NR line, internal hexagon, Korea) and #9, #10, #11, #13, and #14 (Dentium/Implantium, internal hexagon, Korea). Also in mandible, three fixtures were inserted in place of teeth #20 (Dentium/Implantium, internal hexagon, Korea) and #29 and #20 (Dentium/NR line, internal hexagon, Korea). Immediately after surgery, tissue conditioner (TC) (COE-COMFORT, GC, America) was applied in the intaglio surface of IIO (Figure 3(a)). During the healing period, TC was replaced weekly. Six months after implants insertion, second stage surgery was performed and healing abutments were placed. IIO was relieved in place of healing abutments and TC was applied again (Figure 3(b)). Two weeks later, primary impressions with irreversible hydrocolloid (Kerr, USA) were taken. Custom trays were fabricated and open-tray impression copings were placed, hand tightened, and verified with periapical X-ray for proper seating. After border-molding the intact maxilla and then the defect area, open-tray final impression was made with soft putty and light body AC silicon (Panasil, Kettenbach, Germany) simultaneously. The trial record bases were fabricated and bite registration record was taken. In the next appointment, the teeth arrangement were tried-in. The interocclusal-space (IOS) and buccolingual distance were measured on the casts with periodontal probe by using the putty-silicon-index. The decision was to fabricate five-unit implant-supported FPD in anterior segment due to excessive maxillary protrusion. A bar and three ball attachments were used in the maxillary posterior segment. Angled abutments (15° angled abutments, Dentium, Korea) for implant-supported FPD on #7, #9, and #10 were used. Castable abutments (Metal Direct Casting abutment, Dentium, Korea) of the same implant system were used for bar fabrication on #11, #13, and #14. Associated ball and bar attachments (Rhein83 Srl, USA) were selected (Figure 4). Metal framework of implant-supported FPD and bar and ball attachment were waxed up according to the putty index to remain within the contour of the maxillary obturator and then surveyed in a determined path of insertion. Rest seats were prepared on wax up of FPD abutments and guide plans were determined. Superstructures and metal frameworks were tried-in for passive fitness and have been checked with periapical X-ray. Porcelain was applied on metal frames of FPD. Because of excessive IOS, pink porcelain was used in the cervical to simulate the gingiva (FP3). The acrylic denture teeth were arranged on definitive denture base. FPD was surveyed again to determine optimal undercuts for retention of ISO. Teeth arrangement and the FPD were tried-in. After patient's acceptance, a closed-hollow-bulb ISO was processed and the FPD was glazed. At the delivery appointment, the abutments and superstructures were seated and torqued according to the manufacturer's instruction (25–30 NCm). The clinical elastic O-rings were placed inside the metal housings of the obturator. FPD was cemented temporarily (TempBond, Kerr, USA) and ISO was inserted in the patient's mouth simultaneously (Figure 5). In order to make functional impression at the end of delivery appointment, TC (COE-COMFORT, GC, America) was used to improve the peripheral seal and consequently speech ability (Figure 6). The patient was instructed to use the ISO day and night and only remove it for oral hygiene. In the mandible, implant-supported FPD and crown were selected (Figure 7). A soft guard was fabricated on the maxillary FPD (Figure 8). It should be used whenever the ISO is removed for cleaning or changing the caps. After 7 days, the sore spots were relieved off the ISO and TC was applied again for another week. Two weeks after delivery, the patient was satisfied and there was not any traumatic ulcer. ISO with applied-tissue-conditioner was poured and processed again (Figure 9). Fortunately, during one-year follow-up, patient did not have any discomfort and speech problem. The caps were changed every 6 months.

## 3. Discussion

Successful reconstruction of speech and swallowing after maxillectomy relies on using maxillary obturator [7]. Favorable rehabilitation should be planned at the time of tumor surgery in order to design the obturator ideally. Mobility of conventional obturator results in disability in function. In dentate patients, these requirements are achieved by using the remaining teeth, their retentive undercuts, and supportive area of defect. However, fabrication of conventional maxillary obturator may be more challenging in edentulous patients, because the obturator may display different amounts of movement depending on the amount and contour of residual palatal shelf, height of residual alveolar ridge, size of defect, and existing undercuts [6]. To overcome these problems, use of osseointegrated implants is a suitable method for rehabilitation of such patients [8]. In the current patient, because of Angle class II div I profile due to maxillary protrusion, it was not possible to use conventional ISO, because conventional ISO in class II division 1 patients will result in excessive lip protrusion, anesthetic appearance, lack of anterior teeth contacts, and subsequent mandibular anterior-teeth-supraeruption. On the other hand, with reduction of labial flange thickness in this area, shadow of metal framework would be shown through. So an implant-supported FPD in anterior segment was fabricated. In posterior segment, balls and bar substructure were fabricated to provide more retention, support, and stability. Different bar designs and attachments may affect retention and clinical performance of implant-retained obturators [9]. According to Beumer [5], implant-assisted tissue bar designs are preferred for hemimaxillectomy patients. In such design, support is obtained from residual denture-bearing surfaces and key areas of defect, while implants are used to primarily provide stability and retention but not support. According to Ochiai et al. and Assunção et al. [10, 11], implants splinted to each other with a bar transferred less detrimental stress to the implants than individual systems. Celik and Uludag [8] stated that implants splinted with a bar associated with O-rings improve stress distribution compared to the bar clip system. Indeed, the O-ring-type attachment resulted in more favorable stress distribution than either the bar clip or the bar-ERA design. However, the O-ring designs were not as retentive as the other examined attachment systems [5]. By using bar design, positioning the attachments at the same angulation and the same occlusal height is easier. In this way the retention of DO may be improved.

In the mandible, implant-supported FPD and crown were preferred. Mandibular fixed restorations apply more detrimental forces to the maxillary obturator and may compromise its retention, support, and stability [5]. In order to overcome these problems, six implants had been inserted to retain and support the obturator. We paid more attention to the occlusion and stress distribution between defect portion and intact portion of DO. By meticulous selective grinding of acrylic teeth, occlusal contacts were lighter in the defect side. By using mandibular-implant-supported restorations, the patient would not pay attention to the mandibular-removable-prosthesis concurrent with the maxillary obturator. An implant-retained RPD would have replaced all mandibular missing teeth. But we prefer not to replace the mandibular left second molar.

Closed-hollow-bulb-design was selected for this patient. This has some advantages including decreasing weight, preventing retention of nasal secretion, and foods in the bulb and reducing air space in defect. However, it is suggested that oral hygiene and speech intelligibility are improved by open design [12], although our patient did not have these problems.

An ideal obturator should be comfortable, solve speech difficulty, prevent nasal reflux, restore masticatory function, and provide esthetic. In order to provide speech ability and adequate resonance, an appropriate peripheral seal is essential and obturator should prevent air escape from maxillary defect [13]. Functional impression method was used in the presented case in order to achieve these goals.

## 4. Conclusion

Full mouth reconstruction of hemimaxillectomy patients with excessive maxillary protrusion is challenging. Conventional ISO in these patients results in several problems, because it would cause excessive upper lip protrusion, lack of anterior teeth contact, and anesthetic appearance. Using a maxillary anterior FPD along with ISO is preferred in this situation and cause satisfactory results.
